# A confirmation of the predictive utility of the Antibiotic Use Questionnaire

**DOI:** 10.1186/s12889-024-18901-3

**Published:** 2024-07-18

**Authors:** Sebastien Miellet, Mitchell K. Byrne, Nina Reynolds, Taylor Sweetnam

**Affiliations:** 1 School of Psychology, University of Wollongong, Building 41, Northfields Ave, Wollongong, NSW 2522 Australia; 2 Wollongong Antimicrobial Resistance Research Alliance (WARRA), Wollongong, NSW Australia; 3 School of Business, University of Wollongong, Wollongong, NSW Australia; 4 Faculty of Health, Charles Darwin University, Northern Territory, Australia

**Keywords:** Antibiotic, Antibiotic use, Antimicrobial resistance, Attitude, Behaviour change, Social theory, Public health, Measurement, Psychometrics, Cross-cultural

## Abstract

**Background:**

The change in the efficacy of antimicrobial agents due to their misuse is implicated in extensive health and mortality related concerns. The Antibiotics Use Questionnaire (AUQ) is a theory driven measure based on the Theory of Planned Behaviour (TpB) factors that is designed to investigate drivers of antibiotic use behaviour. The objective of this study is to replicate the factor structure from the pilot study within a similar Australian confirmation cohort, and to extend this through investigating if the factor structure holds in a Chinese-identifying cohort.

**Methods:**

The AUQ was disseminated to two cohorts: a confirmation cohort similar to the original study, and a Chinese identifying cohort. Data analysis was completed on the two data sets independently, and on a combined data set. An orthogonal principal components analysis with varimax rotation was used to assess the factor structure, followed by general linear models to determine the influence of the TpB factors on reported antibiotic use.

**Results:**

370 participant responses from the confirmation cohort, and 384 responses from the Chinese-identifying cohort were retained for analysis following review of the data. Results showed modest but acceptable levels of internal reliability across both cohorts. Social norms, and the interaction between attitudes and beliefs and knowledge were significant predictors of self-reported antibiotic use in both cohorts. In the confirmation cohort healthcare training was a significant predictor, and in the Chinese-identifying cohort education was a significant predictor. All other predictors tested produced a nonsignificant relationship with the outcome variable of self-reported antibiotic use.

**Conclusions:**

This study successfully replicated the factor structure of the AUQ in a confirmation cohort, as well as a cohort that identified as culturally or legally Chinese, determining that the factor structure is retained when investigated across cultures. The research additionally highlights the need for a measure such as the AUQ, which can identify how differing social, cultural, and community factors can influence what predicts indiscriminate antibiotic use. Future research will be required to determine the full extent to which this tool can be used to guide bespoke community level interventions to assist in the management of antimicrobial resistance.

**Supplementary Information:**

The online version contains supplementary material available at 10.1186/s12889-024-18901-3.

## Background

The misuse and overuse of antibiotic agents designed for fighting bacterial infections has contributed to antibiotics becoming less effective over time [1–4]. This phenomenon is known as antimicrobial resistance (AMR), and in 2019 it accounted for 4.95 million deaths associated with drug resistant infections, with only ischaemic heart disease and stroke accounting for more deaths in that year [3]. Due to the resistance to (and shortage of) effective antimicrobials, medical issues requiring hospitalisation will require more aggressive treatments, and treatments that are underpinned by antimicrobial drugs (e.g., caesarean sections, hip replacements, gut surgery, or chemotherapy) may become too dangerous to perform [4]. Therefore, while it is estimated that AMR could be responsible for 10 million preventable deaths per year by 2050, O’Neill (2016) reports that this estimate is likely conservative. This situates AMR as one of the leading threats to healthcare globally [3, 4].

Since the COVID-19 pandemic, an increase in antimicrobial resistance has been observed, and has been considered likely attributable to an excessive use of antimicrobial agents throughout the course of the pandemic [5, 6]. Along with mask wearing, good hand hygiene, and social distancing practices, distressed consumers increased their rate of self-medication with antibiotics, for the purpose of protecting themselves against the coronavirus [6]. The prescription of broad-spectrum antibiotics was additionally increased, with one review reporting that 72% of hospitalised COVID-19 patients received antimicrobial therapy, despite only 8% of patients reporting to have experienced bacterial or fungal coinfection [7]. Given this increase in antibiotic use and misuse over the last several years, continued research into the contributors to AMR is imperative.

Previous responses to AMR have invested in public health campaigns aimed at increasing awareness on the issue, for the purpose of supporting positive behaviour change [8]. However, studies have demonstrated that these campaigns have not been associated with desired changes to the public’s understanding of AMR, and the appropriate use of antibiotics [9–11]. These reports align with the other studies examining the effects of public awareness campaigns on behaviours such as promoting healthy eating and physical exercise [12, 13], alcohol intake [14], and tobacco smoking [15]. Toral and Slater (2012) assessed the effects of printed educational materials distributed monthly for a period of six months and concluded that the intervention was ineffective in changing dietary behaviour [13]. Beaudoin et al., (2007), utilised a higher frequency public awareness campaign using radio and television advertisements, and despite an increase in consumers understanding of and positive attitudes towards health behaviours, they reported that there was insufficient evidence of the desired behavioural change [12]. While AMR awareness campaigns have been associated with a significant reduction in prescribing rates [8], studies claim that this reduction in rates of prescription does not influence public awareness or perception of appropriate antibiotic use [16]. Moreover, some studies report that such campaigns have been demonstrated to potentially increase the patient demand for antibiotics in those with poor AMR awareness [17, 18]. Such a paradox, where the impact of a health campaign can produce the opposite of the intended effect [19, 20], could be explained by fear-appeal messages backfiring when people do not believe they can protect themselves from a threat, according to a meta-analysis on threatening communication [21].

One of the more effective programs aimed at targeting AMR is the Swedish Strategic Programme for the Rational Use of Anti-Microbial Agents and Surveillance of Resistance Program (STRAMA), established in 1994 [22]. This multidisciplinary program comprises of several fields including primary care, hospital care, nursing homes, and day-care centres [23]. STRAMA aims to analyse trends in AMR and identify priority settings for interventions. At a local level, the program utilises a bottom-up approach to provide feedback to prescribers and arrange local awareness campaigns for the public [23]. A review of the program by Molstad et al. (2008) indicated that it resulted in a 15% decrease in antibiotic use between 1995 and 2004, and that non-prescribed use of antibiotics is much lower in Sweden compared to neighbouring countries [24]. The review concluded that the coordination across disciplines were important factors in the reduced misuse of antimicrobials [23]. Should information be readily available regarding community level consumer attitudes towards indiscriminate antibiotic use, the STRAMA model could likely be replicated cross-culturally. The bottom-up community level approach is supported by research that demonstrates that despite public awareness campaigns, consumer understanding of AMR did not change substantially between surveys from 2003 to 2008/2009, with even fewer respondents in surveys in 2017 being aware of delayed antibiotics than those in 2014 [9]. Further to this, in a study of 864 participants, only 12.2% knew of the term ‘drug resistance, 6% knew ‘antimicrobial resistance’, and only 5.9% had heard of the term ‘superbug’ [9].

The recent 2020 Australian strategy also emphasised the need to utilise expert views from stakeholders across disciplines for the best combative approaches to AMR, including professionals, the research community, and the public [1]. This strategy recognises that governing the use of antimicrobials through professional sectors and stewardship practices is imperative to protect against unhelpful practices such as the attainment of antibiotics without prescriptions [25]. What this strategy also recognises is that understanding consumer level attitudes and knowledge about the indiscriminate use of antimicrobial agents is crucial to understand patterns of consumption and to design effective interventions [26, 27].

Of consumers that are aware of AMR and the use of antibiotics, there is still widespread misunderstanding of the purpose of antibiotics, with studies reporting rates of up to 54% of participants believing that antibiotics could kill viruses [9], and other studies reporting that only 23.8% of participants were aware that antibiotics are not effective against viruses [28]. Mallah et al. (2020) reported that participants who believed that antibiotics were effective against viruses were two times as likely to engage in any other form of antibiotic misuse behaviour, such as using antibiotics without a prescription, or altering dosage without medical advice [29]. In the same study, 75% of participants expected to receive antibiotics for cold symptoms, a finding reinforced across the literature regarding consumer beliefs that antibiotics will be effective for symptoms of cold and flu [6, 28, 30]. Other problematic behaviours related to antibiotic use for consumers includes retaining unused antibiotics to use in the future, lending unused antibiotics or obtaining antibiotics from friends, obtaining antibiotics without a prescription, seeking a prescription from an alternative doctor when denied a prescription, doubling their dosage, using antibiotics as a preventative measure, or stopping their antibiotic treatment when they feel better [6, 9, 28, 29, 31]. When consumers do choose to dispose of unused antibiotics, they demonstrate challenges understanding the appropriate method of disposal of unused antibiotics, evidenced by reports of flushing leftovers [28]. This contribute to antibiotics in the water system, and is associated with microbes mutating into resistant pathogens, further promoting AMR [28].

In addition to consumer misuse effects, research has demonstrated that there are also significant consumer effects on physician prescribing [30]. Wang (2020) investigated the ways in which caregivers place pressure on physicians prescribing antibiotics in a Chinese paediatric primary care sample [30]. Their findings refute previous concerns that the overprescribing of antibiotic prescriptions in China were due to the incentive structure of physician’s payment schemes, arguing that patients and caregivers are highly influential on the outcome due to frequent advocating for prescriptions of antibiotics. Acquiescence to these requests is a compounding factor in maintaining the normative expectations for antibiotics to be prescribed for minor conditions such as cold and flu [30]. Wong et al. (2021) additionally reported that the pressure placed on physicians to prescribe by patients was related to low level doctor-patient trust in the Chinese community [28]. One of the major factors in the reduction of antibiotic misuse and unnecessary prescription is the trust held by the consumer in the advice provided by their physician [29, 32]. There is a need to understand how the knowledge, attitudes, and practices of antibiotic use vary from one community to another based on the socio-cultural differences between groups [16, 28, 33, 34].

Particularly concerning is the large number of consumers that report accessing antibiotics without prescriptions, whether this be through community pharmacies or online [25]. Gong et al. (2019) found that 79.1% of their participants accessed antibiotics without valid prescriptions through online pharmacies, and 86% through community pharmacies in China [25]. Of the 174 online pharmacies in this study that agreed to dispense antibiotics without a valid prescription, only one explained dosage, and two explained possible adverse reactions [25]. None of the pharmacies explained the duration of the antibiotic course [25]. There is many consumer factors related to indiscriminate antibiotic use, including for instance risk perception [35, 36]. These factors vary considerably based on the individual, social, and cultural factors of users, it is imperative to understand how indiscriminate antibiotic use can be predicted, to effectively implement the recommended bottom-up approach to intervention design [26, 27].

Several factors including attitudes and beliefs related to the use of antibiotics [16], the opinions of others [37], the perceived ability to obtain [25, 28], and a consumer’s knowledge about antibiotics and AMR [6, 16] have been consistently demonstrated to contribute to predicting whether a consumer would use antibiotics indiscriminately. These factors align closely with the Theory of Planned Behaviour (TpB; [38], a model that has been used to predict a range of other health related behaviours [39–42]. The theory posits that a consumer’s behaviour can be predicted by their behavioural intentions [38]. Behavioural intentions are further influenced by a consumers positive or negative attitude towards the behaviour, beliefs about the social expectations of others (subjective norms), and the considerations of the presence of facilitating or impeding factors related to engagement with the behaviour (perceived behavioural control) [39]. Furthermore, research has supported knowledge about the behaviour to be a significant moderating factor in the model across several constructs including the adoption of energy efficient home appliances [43], the use of drone food delivery services [44], household waste sorting [45], and the choice of safer holiday destinations post COVID-19 [46]. An extended TpB model including knowledge as a moderating factor is therefore situated to provide an opportunity to create a theory-based measure for predicting consumer behaviours related to indiscriminate antibiotic use.

Based on these findings, Byrne et al., (2019) designed the Antibiotic Use Questionnaire (AUQ) using the TpB as an underlying structure to predict factors that influenced consumers intentions to obtain and use antibiotics, for the purpose of generating targeted intervention programs at the community level [47]. The TpB factors suggest that a consumer’s antibiotic use behaviours would be influenced by their attitudes towards indiscriminate antibiotic use, the social norms in their community regarding indiscriminate antibiotic use, and their perceived behavioural control regarding the ease of access to antibiotics, extending the theory to include knowledge as a moderating variable [47].

To construct the questionnaire, a multidisciplinary panel of experts from fields including psychology, business, and heath were consulted. Following analysis of 293 responses, eighteen items of the questionnaire were retained that reflected the three variables of the TpB, the outcome variable of behaviour and the covariate of knowledge. The results indicated that antibiotic use behaviour could be significantly explained by each of the variables, and that the TpB model explained 70% of the variance in antibiotic use and misuse.

The AUQ factor structure outcomes were extremely promising and warrant additional investigation to determine if this result can be replicated, and under what circumstances the factor structure holds. Thus, the aim of the current study is the replicate the factor structure from Byrne et al., (2019) within a similar Australian confirmation cohort [47]. Should the factor structure be confirmed, the study then seeks to investigate whether the factor structure remains the same in a Chinese-identifying population cohort to assess for potential cultural biases, and to determine if the factor structure holds cross-culturally.

## Methods

### Data collection and ethics

The first study distributed the AUQ to a similar population to the study conducted by Byrne et al., (2019), with the second study distributing the questionnaire to a population who identified as being of Chinese culture [47]. Panel participants were recruited from March to May 2020. All data was collected online through Qualtrics, and only complete surveys were returned to the authors for analysis. Tacit consent was assumed by virtue of the participants completing and returning the anonymous questionnaire. All responses were anonymous and could not be linked to specific individuals. The studies were approved by the Health and Medical Human Research Ethics Committee (Joint University of Wollongong and Illawarra Shoalhaven Local Health District, 2018/330). The study was performed in accordance with the ethical standards as laid down in the 1964 Declaration of Helsinki and its later amendments or comparable ethical standards.

### Sample

The inclusion criteria for the study were limited to adults of 18 years or older living in Australia in both studies. The first study was additionally restricted to people in the Illawarra region (identified by self-reported postcode), and the gender (50:50) and age demographics were pre-specified as part of a quota sample. In the second study, participants were required to identify as legally (i.e., via citizenship) or culturally Chinese. Of the 433 participants that completed questionnaires for the first study, 10 participants (2.31%) were excluded from the analysis, due to concerns regarding the accuracy and reliability of their responses, after scoring equal to or higher than five in social desirability. An additional 53 participants (12.24%) were excluded as their answers did not show any variance, indicating the same response to all questions. A final sample of 370 participant responses were retained for analysis.

Of the 395 participants that completed questionnaires in the Chinese-identifying cohort, 10 participants (2.53%) were excluded from the analysis, due to concerns regarding the accuracy and reliability of their responses, after scoring equal to or higher than five in social desirability. One participant (0.25%) from Hong Kong was excluded as they indicated they did not identify as culturally Chinese. A final sample of 384 participant responses were retained for analysis.

### Measures

The antibiotic use questionnaire (AUQ) is a 30 item self-report measure designed to assess factors influencing community antibiotic use and misuse [47]. The sociodemographic section of the questionnaire includes six questions regarding the respondents age, gender, educational attainment, residence, training in health-related fields, and presence of a healthcare worker in their family or friend group. The 18 response items (Table 1) related to antibiotic use are anchored on a 4-point Likert scale (i.e., ‘antibiotics will reduce my cold symptoms’), and are based on the three variables of the TpB (attitudes, subjective norms, and perceived behavioural control), the outcome variable (intended behaviour), and the covariate (knowledge). Six items intended to measure social desirability from the Marlowe-Crowne Social Desirability Scale [48] are anchored with a dichotomous true/false response style. The sociodemographic section was presented first then the antibiotic-use and social-desirability items were presented in a random order. AUQ scores have been normalised to have a 0 as a minimum score with a 10 as a maximum score, with low scores being reflective of less rational antibiotic use, and high scores reflecting rational antibiotic use [47].

**Table 1:** Item number and corresponding items

Item Number	Item
Item 1.	Antibiotics will reduce my cold symptoms
Item 2.	My friends and family follow recommendations for antibiotic use
Item 3.	Antibiotics are needed for the common cold
Item 4.	Antibiotics may have negative side effects
Item 5.	I would take antibiotics without consulting a doctor
Item 6.	I use leftover or unused antibiotics or scripts
Item 7.	It is my right to ask for an antibiotic from my doctor
Item 8.	My friends and family only use antibiotics when prescribed
Item 9.	I know I need antibiotics before I see my doctor
Item 10.	In my community it is common to use antibiotics without a prescription
Item 11.	I feel confident to ask for antibiotics when I need them
Item 12.	Antibiotics are less likely to work in the future
Item 13.	I consult with my doctor prior to taking antibiotics
Item 14.	I keep leftover or unused antibiotics or scripts
Item 15.	I could easily get antibiotics from a doctor
Item 16.	I could easily get antibiotics online
Item 17.	I could easily get antibiotics from my family / a friend / household
Item 18.	By the time I am sick enough to see my doctor, I expect a prescription of antibiotics

### Data analysis

Data analysis was carried out using Matlab R2018A (The MathWorks Inc) and Jamovi. The analytical procedures were identical to those described in Byrne et al. (2019) [47]. All analyses were run for each data set independently, and then again on the combined dataset to include the cultural background in the analysis. The process of the analysis was as follows. First the initial screening of responses to remove those that included missing data, outliers and or coding errors. Second, descriptive statistics were reported. Third, we used an orthogonal principal components analysis with varimax rotation to check the factor structure. Forth, we determined the internal reliability of the factors. Finally, general linear models were used to determine the influence of the TpB factors on reported antibiotic use.

## Results

### Confirmation cohort

The participants were predominantly female (58%; *n =* 214), with 27% reporting having a bachelor degree qualification or higher (*n* = 99), and ages ranging from 18 to 84 years (*M* = 51.00, *SD* = 18.00). Most respondents were not personally trained in a health-related field (80%; *n =* 298) and did not have a family member or friend with a health-related occupation 71% (*n* = 262).

Factor loadings of the questionnaire items for the three variables of the TpB, the outcome variable (behaviour), and covariate (knowledge), are reported in Table 2. All five variables encompassed four items except for social norms, which included two items, yielding a final 18-item questionnaire.

**Table 2:** Factor loading for questionnaire items (Confirmation cohort) *

Item Number		Behaviour	PBC	Knowledge	Attitudes and Beliefs	Social Norms
Item 1.		0.053	0.197	**0.691**	0.129	0.067
Item 2.		0.088	0.090	-0.017	0.029	**0.663**
Item 3.		0.082	0.177	**0.739**	-0.002	0.063
Item 4.		0.140	0.064	**0.342**	0.059	-0.026
Item 5.		**0.583**	0.163	0.203	0.186	0.331
Item 6.		**0.825**	0.050	0.154	0.163	0.114
Item 7.		0.072	0.000	0.278	**0.597**	0.049
Item 8.		0.202	0.278	0.038	0.024	**0.637**
Item 9.		0.228	-0.022	0.173	**0.516**	0.033
Item 10.		0.236	**0.474**	0.268	0.055	0.225
Item 11.		0.084	0.085	0.017	**0.595**	-0.029
Item 12.		0.056	-0.133	**0.417**	0.074	0.0175
Item 13.		**0.464**	0.146	0.243	-0.048	0.422
Item 14.		**0.702**	0.210	0.117	0.265	0.085
Item 15.		-0.0344	**-0.337**	0.057	*-0.403*	-0.038
Item 16.		-0.022	**-0.478**	-0.037	-0.051	-0.133
Item 17.		-0.190	**-0.749**	-0.097	-0.059	-0.098
Item 18.		0.208	0.059	*0.538*	**0.405**	0.067

*Bold font indicates which factor the items are associated with; italics indicates that the highest factor loading is different from the factor the item is associated with

Results showed modest but acceptable levels of internal reliability (Cronbach alpha) within each variable: attitudes and beliefs = 0.68; social norms = 0.67; PBC = -0.05; knowledge = 0.64; behaviour = 0.82. The low Cronbach alpha for PBC is not unexpected as this factor does not measure a homogeneous construct. Indeed, the items forming this factor investigate potential sources for antibiotics (i.e., “I could easily get antibiotics online / from a doctor / family / a friend / household)” that do not necessarily correlate. Thus, these items can be considered separately. Those items will be detailed in conjunction with the results from the Chinese-identifying cohort.

The factor analysis was run again without the items 10, 15, 16 and 17 which were associated with PBC, and factor loadings for this analysis are reported in Table 3.

**Table 3:** Factor loading for questionnaire items: Excluding items related to PBC(Confirmation cohort)*

Item Number		Behaviour		Knowledge		Attitudes and Beliefs		Social Norms
1.		0.074		**0.773**		0.175		0.153
2.		0.037		-0.026		0.065		**0.862**
3.		0.125		**0.783**		0.084		0.115
4.		0.242		**0.552**		-0.065		-0.155
5.		**0.730**		0.131		0.217		0.236
6.		**0.833**		0.143		0.161		0.072
7.		0.041		0.280		**0.698**		0.064
8.		0.274		0.045		-0.035		**0.758**
9.		0.263		0.103		**0.679**		-0.028
11.		0.097		-0.079		**0.778**		-0.041
12.		0.026		**0.618**		0.092		-0.033
13.		**0.556**		0.202		-0.105		0.450
14.		**0.800**		0.110		0.226		0.047
18.		0.211		0.506		**0.494**		0.104

*Bold font indicates which factor the items are associated with

A general linear model was run with the following fixed effects: the interaction between attitudes and beliefs and knowledge; social norms; age; gender; education; were health trained, had a health worker in the family. Fixed effects coefficients can be found in Table 4. For this model the Akaike Information Criterion (AIC) was 1572.1 and the Bayesian Information Criterion (BIC) was 1611.3.

**Table 4:** Fixed effects coefficients (Confirmation cohort)

Name	Estimate	Standard Error	t-Statistic	Degrees of Freedom	*p*-Value	η^2^*p*
Intercept	3.222	0.998	3.227	363	0.001	0.028
Interaction between attitudes, beliefs and knowledge	0.031	0.003	9.402	363	6.1791e-19	0.196
Social norms	0.325	0.046	7.029	363	1.0389e-11	0.120
Age	-0.002	0.006	-0.303	363	0.762	0.000
Gender	0.245	0.208	1.178	363	0.240	0.004
Education level	0.082	0.090	0.911	363	0.363	0.002
Personal training in a health-related field	-0.893	0.292	-3.055	363	0.002	0.025
Family member or friend with a health-related occupation	0.050	0.259	0.195	363	0.845	0.000

The ordinary R-squared was 0.742 and the adjusted R-squared was 0.737; indicating that this model explains more than 70% of the variance in the self-reported antibiotic misuse. The fixed effect variables Social Norms (β = 0.33, *p* < .001), interaction between attitudes and beliefs and knowledge (β = 0.03, p = < 0.001), and the training in healthcare (β = − 0.89, *p* < .003), were all significant predictors of antibiotic use behaviour. All other predictors tested did not produce a significant relationship with the outcome variable.

### Chinese cohort

The participants were predominantly female (60%; *n =* 230), with 76% reporting having a bachelor degree qualification or higher (*n* = 293), and ages ranging from 18 to 74 years (*M* = 38.00, *SD* = 13.00). Most respondents were not personally trained in a health-related field (78%; *n =* 299) and did not have a family member or friend with a health-related occupation 71% (*n* = 272).

Factor loadings of the questionnaire items for the three variables of the TpB, the outcome variable (behaviour), and covariate (knowledge), are reported in Table 5. All five variables encompassed four items except for social norms, which included two items, yielding a final 18-item questionnaire.

**Table 5:** Factor loading for questionnaire items (Chinese-identifying cohort cohort) *

Item Number	Behaviour	PBC	Knowledge	Attitudes and Beliefs	Social Norms
Item 1.	0.05	-0.13	**0.75**	0.22	0.04
Item 2.	0.06	0.04	0.12	0.22	**-0.32**
Item 3.	0.18	-0.10	**0.68**	0.25	0.17
Item 4.	-0.20	-0.01	**-0.20**	0.00	-0.08
Item 5.	**0.34**	-0.18	0.31	0.22	*0.59*
Item 6.	**0.83**	-0.11	0.26	0.15	0.17
Item 7.	0.12	-0.22	0.18	**0.60**	-0.05
Item 8.	-0.15	0.29	0.03	0.03	-0.43
Item 9.	0.16	-0.12	0.25	**0.40**	0.26
Item 10.	0.20	**-0.16**	0.15	0.18	*0.47*
Item 11.	0.08	0.00	0.04	**0.62**	0.01
Item 12.	-0.09	-0.06	**-0.29**	-0.02	0.01
Item 13.	**-0.12**	0.16	-0.10	0.00	*-0.50*
Item 14.	**0.58**	-0.09	0.12	0.25	0.24
Item 15.	0.03	**0.42**	0.03	-0.19	-0.04
Item 16.	-0.06	**0.44**	-0.06	0.05	-0.18
Item 17.	-0.13	**0.65**	-0.01	-0.04	-0.33
Item 18.	0.07	0.05	0.39	**0.47**	0.00

*Bold font indicates which factor the items are associated with; italics indicates that the highest factor loading is different from the factor the item is associated with

Results showed modest but acceptable levels of internal reliability (Cronbach alpha) within each variable: attitudes and beliefs = 0.65; social norms = 0.40; PBC = 0.13; knowledge = 0.60; behaviour = 0.74. As before, the low Cronbach alpha for PBC is not unexpected as this factor does not measure a homogeneous construct, given that they investigate potential sources for antibiotics that do not necessarily correlate. Thus, these items can be considered separately. Those items will be detailed in conjunction with the results from the confirmation cohort.

As per the confirmation cohort, the factor analysis was run again without the items 10, 15, 16 and 17 which were associated with PBC, and factor loadings for this analysis are reported in Table 6.

**Table 6:** Factor loading for questionnaire items: Excluding items related to PBC(Chinese-identifying cohort)*

Item Number	Behaviour	Knowledge	Attitudes and Beliefs	Social Norms
1.	0.07	**0.75**	0.24	-0.02
2.	0.06	0.10	0.21	**0.37**
3.	0.20	**0.68**	0.27	-0.14
4.	-0.20	**-0.20**	0.01	0.07
5.	**0.38**	0.33	0.26	-0.53
6.	**0.84**	0.25	0.17	-0.15
7.	0.12	0.18	**0.61**	0.00
8.	-0.17	0.00	-0.02	**0.47**
9.	0.18	0.25	**0.43**	-0.22
11.	0.08	0.03	**0.61**	0.05
12.	-0.08	**-0.28**	-0.04	-0.01
13.	**-0.12**	-0.12	-0.05	*0.60*
14.	**0.59**	0.11	0.27	-0.22
18.	0.07	0.37	**0.46**	0.06

*Bold font indicates which factor the items are associated with, italics indicates that the highest factor loading is different from the factor the item is associated with

A general linear model was run with the following fixed effects: the interaction between attitudes and beliefs and knowledge; social norms; age; gender; education; were health trained, had a health worker in the family. Fixed effects coefficients can be found in Table 7. For this model the Akaike Information Criterion (AIC) was 1633.4 and the Bayesian Information Criterion (BIC) was 1672.9.

**Table 7:** Fixed effects coefficients (Chinese-identifying cohort)

Name	Estimate	Standard Error	t-Statistic	Degrees of Freedom	*p*-Value	η^2^*p*	
Intercept	6.149	1.00	6.133	376	2.190	0.091	
Interaction between attitudes, beliefs and knowledge	0.030	0.003	11.364	376	6.3108e-26	0.256	
Social norms	0.255	0.046	5.595	376	4.2607e-08	0.077	
Age	0.001	0.008	0.149	376	0.881	0.000	
Gender	0.243	0.213	1.143	376	0.254	0.003	
Education level	-0.261	0.105	-2.493	376	0.0131	0.016	
Personal training in a health-related field	0.178	0.279	0.640	376	0.523	0.001	
Family member or friend with a health-related occupation	0.048	0.253	0.189	376	0.850	0.000	

The ordinary R-squared was 0.736 and the adjusted R-squared was 0.731; indicating that this model explains more than 70% of the variance in the self-reported antibiotic misuse. The fixed effect variables Social Norms (β = 0.25, *p* < .001) and interaction between attitudes and beliefs and knowledge (β = 0.03, p = < 0.001) are significant predictors of antibiotic use behaviour. In contrast to the confirmation cohort, here the training in healthcare was non-significant (β = − 0.18, *p* = .52) but the education variable was significant (β = − 0.26, *p* < .02). All other predictors tested did not produce a significant relationship with the outcome variable.

#### Combined

The data from the 370 respondents of the confirmation cohort and the 384 respondents from the Chinese cohort were combined (see Appendix 1 for a correlation matrix of all variables). Factor loadings of the questionnaire items for the variables of the TpB (excluding PBC), the outcome variable (behaviour), and covariate (knowledge), are reported in Table 8.

**Table 8:** Factor loading for questionnaire items (Combined cohort) *

Item Number	Behaviour	Knowledge	Attitudes and Beliefs	Social Norms
1.	0.06	**0.77**	0.18	0.08
2.	-0.03	-0.07	-0.08	**0.53**
3.	0.15	**0.72**	0.17	0.14
4.	0.16	**0.24**	0.03	0.02
5.	**0.44**	0.25	0.29	0.44
6.	**0.81**	0.24	0.17	0.19
7.	0.10	0.24	**0.56**	-0.01
8.	0.17	0.05	0.02	**0.55**
9.	0.20	0.18	**0.50**	0.10
11.	0.09	0.02	**0.60**	-0.07
12.	0.08	**0.33**	0.08	-0.01
13.	**0.22**	0.16	0.05	*0.56*
14.	**0.64**	0.17	0.25	0.19
18.	0.09	0.44	**0.46**	0.05

*Bold font indicates which factor the items are associated with; italics indicates that the highest factor loading is different from the factor the item is associated with

The results from the combined cohort showed modest but acceptable levels of internal reliability (Cronbach alpha) within each variable: attitudes and beliefs = 0.67; social norms = 0.52; knowledge = 0.63; behaviour = 0.77.

A general linear model was run with the following fixed effects: the interaction between attitudes and beliefs and knowledge; social norms; age; gender; education; were health trained, had a health worker in the family and cultural background. Fixed effects coefficients can be found in Table 9. For this model the Akaike Information Criterion (AIC) was 3184.2 and the Bayesian Information Criterion (BIC) was 3235.

**Table 9:** Fixed effects coefficients (Combined cohort)

Name	Estimate	Standard Error	t-Statistic	Degrees of Freedom	*p*-Value	η^2^*p*	
Intercept	5.547	0.705	7.864	745	1.303	0.077	
Group	-0.082	0.169	-0.487	745	0.627	0.000	
Interaction between attitudes, beliefs and knowledge	0.028	0.002	14.85	745	6.6374e-44	0.228	
Social norms	0.279	0.032	8.617	745	4.1012e-17	0.091	
Age	0.001	0.005	0.225	745	0.822	0.000	
Gender	-0.003	0.147	-0.023	745	0.982	0.000	
Education level	-0.144	0.068	-2.116	745	0.035	0.006	
Personal training in a health-related field	0.492	0.200	2.458	745	0.014	0.008	
Family member or friend with a health-related occupation	0.054	0.180	0.297	745	0.766	0.000	

The ordinary R-squared was 0.742 and the adjusted R-squared was 0.739; indicating that this model explains more than 70% of the variance in the self-reported antibiotic misuse. The fixed effect variables Social Norms (β = 0.28, *p* < .001) and interaction between attitudes and beliefs and knowledge (β = 0.027, p = < 0.001) are significant predictors of antibiotic use behaviour. This is also the case for training in healthcare (β = 0.49, *p* = .014) and education (β = − 0.14, *p* < .035). All other predictors tested did not produce a significant relationship with the outcome variable.

It is interesting to note that the survey travels well across cultural groups despite significant differences in the samples on level of education and age. It is additionally interesting to note that behaviour, knowledge, attitudes and beliefs, and social norms show significant differences between groups, both with conventional t-tests, and with the robust Yuen test (Table 10). These between group differences may reflects the capacity for the AUQ to provide insight into antibiotic use despite cultural differences related to the factors of the TpB.

**Table 10:** Robust independent samples T-test

Name	t-Statistic	Degrees of Freedom	*p*-Value	Mean difference	Lower	Upper
Education	14.10	336	< 0.001	-1.088	-1.2404	-0.937
Age	11.90	347	< 0.001	16.007	13.3602	18.653
Behaviour	3.65	449	< 0.001	0.757	0.3497	1.164
Knowledge	3.99	441	< 0.001	0.658	0.3334	0.982
Attitudes and Beliefs	1.39	450	0.166	0.202	-0.0843	0.489
Social Norms	2.10	437	0.037	0.400	0.0249	0.775

## Discussion

Byrne et al., (2019) designed the AUQ to assess factors related to antibiotic use and misuse in the community, using the TpB as a theoretical model [47]. The results of the original study indicated that antibiotic use behaviour could be significantly explained by each of the variables, and that the TpB model explained 70% of the variance in antibiotic use and misuse. The aim of the current study was to determine if this result could be replicated in a similar Australian cohort, and to additionally determine if the factor structure holds in a Chinese-identifying population.

The factor analysis identified items corresponding to the three variables of the TpB, the outcome variable (behaviour), and the covariate knowledge. The internal reliability values were acceptable for all variables except for PBC. This is likely because while each item is reflecting the same construct (perceived ability to obtain antibiotics), the items enquire about different sources from whom consumers may obtain their antibiotics, such as friends, doctors, or online. It would therefore be unlikely that there would be a high internal reliability value for these items, given that consumers who believe they could easily get antibiotics from doctors may not necessarily believe that they could easily get them from friends, or through online services.

A linear-mixed effects analysis revealed that across the Australian confirmation cohort, the Chinese-identifying cohort, and the combined cohort, the intent of antibiotic use behaviour can be significantly explained by social norms, and attitudes and beliefs moderated by knowledge. In all cohorts these variables predicted over 70% of the variance in antibiotic use and misuse, which replicated the original study. These findings support the use of the measure in the prediction of indiscriminate antibiotic use.

The demographic predictors varied across the cohorts, with training in healthcare being the only significant predictor in the Australian confirmation cohort. This differs to the original study by Byrne et al., (2019), where the presence of a healthcare worker in the family was a significant demographic predictor [47]. In the Chinese-identifying cohort the significant demographic predictor was level of education, which was not a significant predictor in either the original study or the confirmation cohort. This supports the need for a measure such as the AUQ, given that the results identify varying contributing factors based on the social and cultural features of antibiotic users [26, 27]. For example, McNulty (2007) found that a higher level of education was related to a self-reported belief that participants knew more about antibiotics and were therefore confident in retaining unused antibiotics (a known factor in the increase in AMR) [10]. A targeted intervention for the Chinese-identifying cohort may include a component directly related to this, while the Australian confirmation cohort from study one may include a component related to having a healthcare worker in the family, and from study two the Australian cohort may need a component related to their own training in a healthcare field.

The present study is primarily limited by the results of the PBC variable differing to that of the original study, where PBC demonstrated an acceptable level of internal reliability (Byrne et al., 2019). Though it seems reasonable to state that this is due to the items investigating different sources for obtaining antibiotics, it does not explain why the PBC variable has different internal validity outcomes across studies. Additional investigation into the validity of the PBC items is required and including questions directly related to individuals’ perception of control over the correct use of antibiotics might improve validity. Another factor to consider is the use of a Chinese-identifying cohort. While participants may have identified as culturally Chinese, this does not necessarily indicate that they would behave in a similar way if living in China. Research shows that there are differences in the ease of obtaining antibiotics without a prescription in Australia as compared to China [25], and additionally differences in other factors related to AMR such as physician-patient trust [28]. It may therefore be helpful to obtain a sample from a cohort currently living within the country of interest, to best determine the needs for community level intervention.

While the TpB conceptualises intentions to predict behaviour, it is worth noting that the AUQ Behaviour items cover reported behaviour. Reported behaviour is likely to be more reliable than behavioural intentions as we directly asked the participants what they generally do rather than what they intend to do. However, reported behaviour is not perfectly equivalent to actual behaviour because of phenomena such as wishful thinking, context and conformity. Prescription data are not available for privacy reasons, and it might not predict perfectly antibiotics use. Therefore, reported antibiotic use, despite its limitations is the best proxy of actual behaviour available for this study.

Though replicating the factor structure of the AUQ in a Chinese-identifying cohort is a promising start, future research is required across other international samples to determine if the questionnaire is valid across other cultures. The ability of the measure to inform intervention programs as intended also needs to be tested, through verifying the intentions to use antibiotics and actual antibiotic use behaviour. An assessment of whether an intervention informed by the measure at a community level can be successful is also necessary.

## Conclusions

This study demonstrated that the capacity for the AUQ to predict antibiotic use and misuse was not restricted to the cohort used in the original study, and that the results can be replicated not only in a similar cohort, but cross-culturally. The research additionally highlights the need for a measure such as the AUQ, which can identify how differing social, cultural, and community factors can influence what predicts indiscriminate antibiotic use. These findings have implications for how bottom up, community level approaches to interventions can be effectively designed. With this knowledge, and the bespoke interventions that may come from it, researchers and policy makers can make an impact on the global health threat that is AMR.

### Electronic supplementary material

Below is the link to the electronic supplementary material.

Supplementary Material 1

## Data Availability

The datasets used and/or analysed during the current study are available from the corresponding author on reasonable request.

## Abbreviations

AMR: Antimicrobial resistance
AUQ: Antibiotic Use Questionnaire
AIC: Akaike Information Criterion
BIC: Bayesian Information Criterion
STRAMA: Swedish Strategic Programme for the Rational Use of Anti-Microbial Agents and Surveillance of Resistance Program
PBC: Perceived behavioural control
M: Mean
SD: Standard deviation
TpB: Theory of Planned Behaviour
WHO: World Health Organisation
