# Supplementary figures and images for: A confirmation of the predictive utility of the Antibiotic Use Questionnaire
Source: BMC Public Health. 2024 Jul 18;24:1925. doi: 10.1186/s12889-024-18901-3 (PMC11256407; doi:10.1186/s12889-024-18901-3)

**Appendix 1**


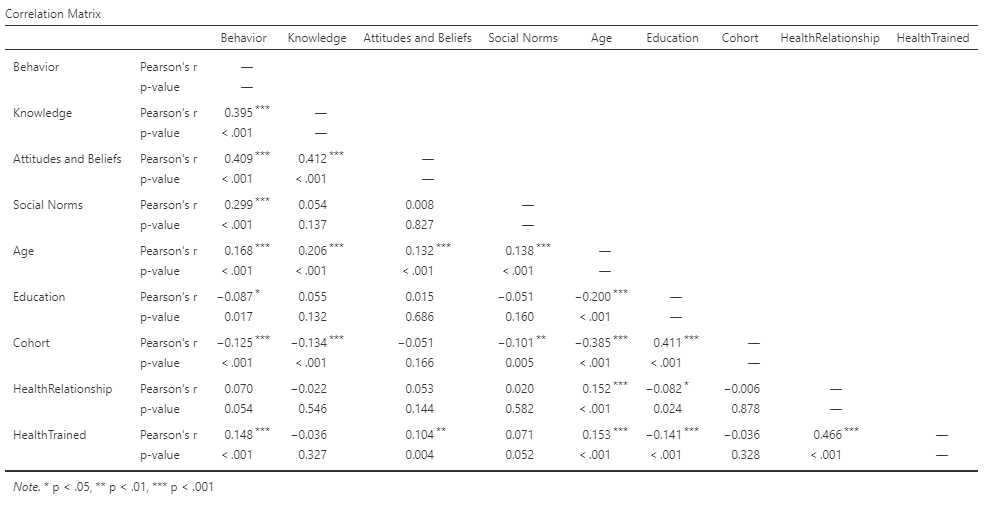

Supplement: Supplementary file 1 — Supplementary Material 1 [file 12889_2024_18901_MOESM1_ESM.docx]
